# Interlink Between Physiological and Biomechanical Changes in the Swim-to-Cycle Transition in Triathlon Events: A Narrative Review

**DOI:** 10.1186/s40798-022-00521-z

**Published:** 2022-10-12

**Authors:** Luca Ambrosini, Valentina Presta, Daniela Galli, Prisco Mirandola, Marco Vitale, Giuliana Gobbi, Giancarlo Condello

**Affiliations:** 1 Department of Medicine and Surgery, University of Parma, Via Gramsci 14, 43126 Parma, Italy; 2 Clinical Movement Analysis Laboratory, University of Parma, 43126 Parma, Italy

**Keywords:** Swimming intensity, Pedal cadence, Gross efficiency, Pacing strategy, Responses

## Abstract

Triathlon is a multisport composed of swim, cycle, and run segments and two transition periods. The swim-to-cycle transition is considered a critical period for the change in body position and the modifications in physiological (heart rate, VO_2_, lactate) and biomechanical parameters (cycling power and cadence, swimming stroke rate). Therefore, the aim of this review was to summarize the current evidence regarding the physiological and biomechanical changes and their interlink during the swim-to-cycle transition hinting at practical recommendations for coaches and athletes. The influence of the swim segment on cycle one is more evident for short-distance events. Greater modifications occur in athletes of lower level. The modulation of intensity during the swim segment affects the changes in the physiological parameters (heart rate, blood lactate, core temperature), with a concomitant influence on cycling gross efficiency. However, gross efficiency could be preserved by wearing a wetsuit or by swimming in a drafting position. A higher swim leg frequency during the last meters of the segment induces a higher cadence during the cycle segment. Training should be directed to the maintenance of a swimming intensity around 80–90% of a previous maximal swim test and with the use of a positive pacing strategy. When athletes are intended to train consecutively only swim and cycle segments, for an optimal muscle activation during cycling, triathletes could adopt a lower cadence (about 60–70% of their typical cadence), although an optimal pedaling cadence depends on the level and type of athlete. Future research should be focused on the combined measurements of physiological and biomechanical parameters using an intervention study design to evaluate training adaptations on swim kick rate and their effects on cycling performance. Coaches and athletes could benefit from the understanding of the physiological and biomechanical changes occurring during the swim-to-cycle transition to optimize the overall triathlon performance.

## Key Points

- In triathlon, the physiological (heart rate, VO_2_, lactate) and/or biomechanical parameters (cycling power and cadence, swimming stroke rate) might vary according to the different postures adopted during the first two segments; hence, the swim-to-cycle transition can be considered a critical period during which the body passes from horizontal to upright position.
- The different race distances can influence the physiological responses, since more power and speed are necessary for short triathlon distances, while longer distances require more endurance and pacing strategy. Therefore, similar to physiological parameters, biomechanical responses can be influenced by race distance, intensity, pacing strategy, and especially wearing the wetsuit.
- The influence of the swimming interpretation on the subsequent cycling segment is more evident for the short-distance events compared to the full-distance triathlons. It is important to propose periodic swim-to-cycle tests for the evaluation of physiological and biomechanical parameters and the determination of an optimal interlink among them.

## Introduction

Triathlon was established in San Diego (USA) in the early 1970s, when the San Diego Track Club organized the first event combining running (10 km), cycling (8 km), and swimming (500 m) disciplines in the same race. The distance for each segment has changed over the years until the debut in the 2000 Olympic Games in Sydney, with the Olympic distance of 1.5-km swim, 40-km bike, and 10-km run. Currently, several race distances are recognized, such as Sprint distance, Olympic distance, Half distance, and Full distance (Table 1). Moreover, the Mixed-team relay is a new short race event during which four athletes (in the order female–male–female–male) complete a super-sprint triathlon [1, 2].

**Table 1:** Triathlon race type, related distance, and duration

Race type	Swim segment (m)	Bike segment (km)	Run segment (m)	Duration range
Mixed-team relay	300	7	2000	15–20 min
Sprint	750	20	5000	50–90 min
Olympic	1500	40	10,000	105–150 min
Half distance	1900	90	21,097	3–6 h
Full distance	3800	180	42,195	7–12 h

The first Full-distance triathlon competition was held in 1978 in Hawaii and today it represents the most famous triathlon competition in the world. Para-triathlon and Mixed-team relay race made their debut in Rio 2016 Paralympic Games and Tokyo 2021 Olympic Games, respectively.

The duration of a race can last 15–20 min for the Mixed-team relay up to 7–12 h for the Full-distance triathlon, while elite Olympic distance athletes can finish a competition in less than 2 h. Triathlon cannot be considered a single performance but as a sequence of the three different disciplines, hence each segment can influence the subsequent one and the overall performance [3], especially for *World Triathlon Series* (WTS) shorter distance events (i.e., Mixed-team relay, Sprint distance, and Olympic distance) [2]. Generally, the contribution of each segment to the total time is up to 20% for swimming, between 50 and 60% for cycling, and 30–40% for running [2]. Although the swim segment is the shortest distance of the race, swimming could have a substantial influence on the final result in Olympic distance [2, 4], as it has been observed that 90% of male and 70% of female winners exit the water in the first pack [4]. Moreover, swim and cycle segments are also considered the foundations for the running performance [5]. During Sprint and Olympic distance events, swimming in the first pack at a lower intensity increases the chance to stay in the first pack during cycling and to preserve energy for the final run segment [6, 7]. Therefore, even though running performance is considered decisive for the overall success [5], a faster swimming time is beneficial for the triathlon performance [8, 9].

A crucial phase of the triathlon performance is the transition period, defined as the final portion of a segment and the initial portion of the subsequent segment. Hence, it is possible to distinguish the swim-to-cycle (T1) and cycle-to-run (T2) transition [1]. An optimal link among the three triathlon disciplines should be achieved to minimize the time loss during the two transitions [10]. T1 lasts longer than T2 and is characterized by a greater variability, because it is influenced by (i) the position of the athlete in the group when she/he enters the transition area, and (ii) the time spent to make the specific transition actions. Therefore, T1 has been thought to affect the physiological conditions of athletes and limit the performance outcome [11, 12].

**Fig. 1:**
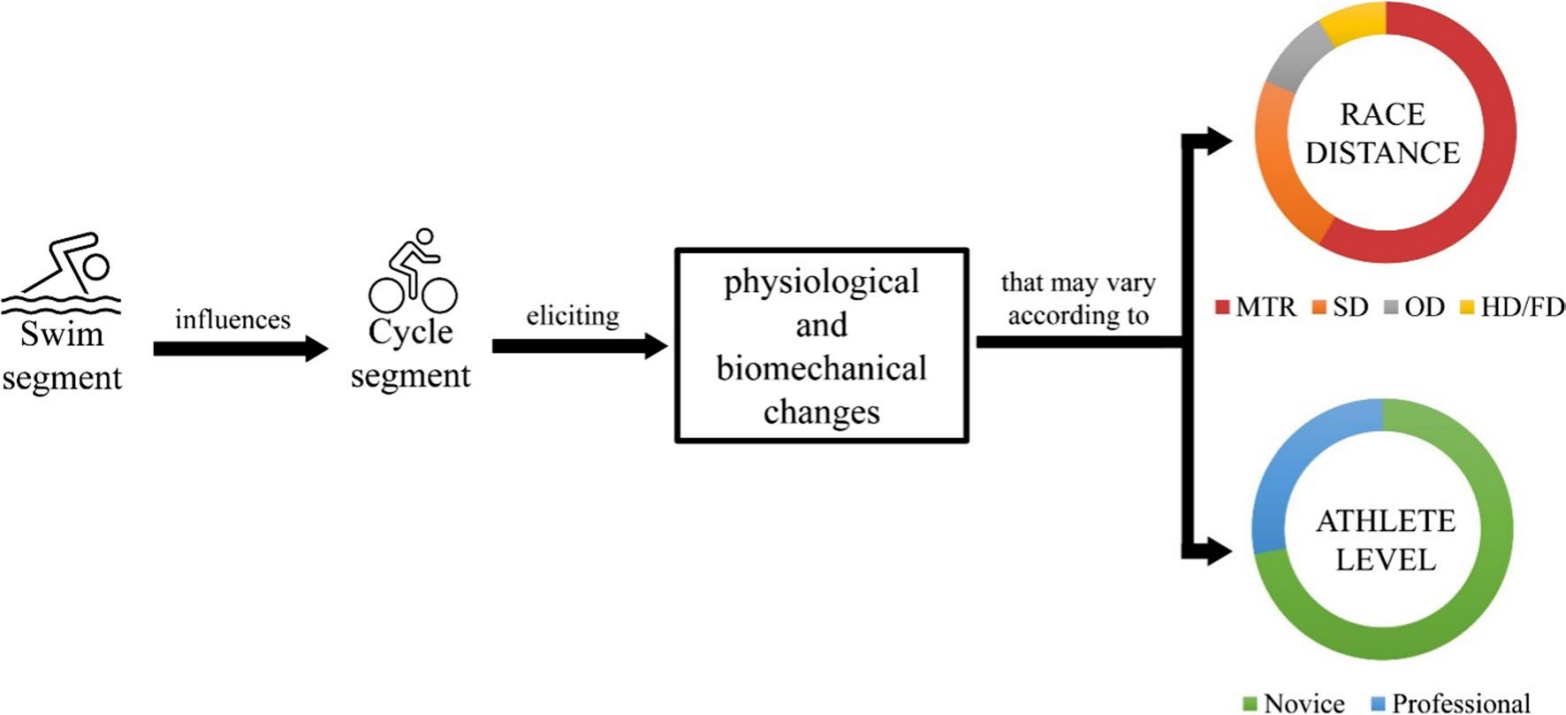
Summary of physiological and biomechanical changes according to race distance and athlete level. Swim segment influences the subsequent cycle one eliciting both physiological and biomechanical changes, which may vary according to race distance and athlete level. The ring charts are nonquantitative interpretations of variables (race distance and athlete level) triggering greater or smaller physiological and biomechanical changes. The colors and size of ring sections are qualitative examples to visually explain the physiological and biomechanical changes during different race distances and according to the athlete level. The top ring chart shows that greater modifications (red, orange, and gray sections) occur in short distances (MTR, SD, and OD), whereas longer distances (HD/FD) elicit fewer physiological and biomechanical changes (yellow sections). The bottom ring chart shows that physiological and biomechanical changes are more evident in novice athletes (green segment) compared to professional athletes (blue segment). *HD* Half distance; *FD* Full distance; *MTR* Mixed-team relay; *OD* Olympic distance; *SD* Sprint distance

The different postures (i.e., horizontal and upright) adopted by athletes during the first two segments might influence the physiological and/or biomechanical responses, highlighting the importance of investigating swim-to-cycle transition. The evaluation of changes in physiological parameters according to a different posture or intensity during exercise has received attention in the past [13–17]. Moreover, the different triathlon distances can induce different responses. In general, the low intensity observed during the swim segment of long events (Full distance) does not cause excessive alteration in the physiological and biomechanical parameters [18]. Conversely, with a decrease in the swim segment distance (from Half distance to Mixed-team relay), the increase in intensity might induce greater physiological and biomechanical changes (Fig. 1) [19]. Therefore, it has been deemed necessary to investigate the physiological and biomechanical changes during the swim-to-cycle transition. However, while the cycle-to-run transition has been widely debated and reviewed [20], a summary of evidence regarding the swim-to-cycle transition is lacking. Therefore, the aim of this review was to summarize the available evidence regarding the physiological and biomechanical changes during the swim-to-cycle transition, hinting at practical recommendations for coaches and athletes. Firstly, the evidence is summarized for the physiological and biomechanical changes in isolation and, secondly, exploring the interlink among both changes. Moreover, the different triathlon distances and factors associated with swimming characteristics (i.e., leg frequency, intensity, pacing strategy) and conditions (i.e., wetsuit) have been debated since they can induce specific responses and further affect the next cycle segment, hence having a positive or negative impact on the entire triathlon performance.

## Physiological Changes

The multidisciplinary nature of triathlon induces a unique physiological load on athletes, with the modification of hemodynamic, biochemical, cardiorespiratory, and metabolic parameters. The different race distances can influence the physiological responses, since more power and speed are necessary for short triathlon distances, while longer distances require more endurance and strategy [21, 22]. In general terms, triathletes show high levels of aerobic capacity, but lower compared to the specialists in the single disciplines, and a greater work economy, with the ability to express a better performance at a lower percentage of maximal aerobic capacity [23–26]. Moreover, triathletes have a high anaerobic threshold and cardiovascular parameters, with an excellent management of effort and energy expenditure, which are considered critical factors for the success during a triathlon competition [23–26].

### Hemodynamic and Biochemical Parameters

The modifications in the plasma volume are mainly due to the postural change (shift from horizontal to upright position) than the intensity of exercise, which, in turn, influences only 1% of plasma volume change [27, 28]. A gradual reduction in plasma volume was observed in novice athletes during the Sprint distance by 3.8% from resting to the end of the swim segment and by 4.3% until the end of the cycle segment [28]. Plasma volume variation is due to the blood volume increment during the horizontal posture as compared to the upright one. Indeed, the supine position allows a more efficient drainage of body fluids in vessels, increasing blood volume. However, concurrent factors can be responsible for the modification in the plasma volume, such as the hydration status, posture, exercise intensity, and water/body temperature. Indeed, with maximal and submaximal endurance performance, the increase in the internal temperature and the dehydration compromise muscle aerobic metabolism [13, 15, 17, 29, 36]. Moreover, hyperthermia and dehydration lead to a reduction in stroke volume and a consequent increase in heart rate (i.e., *cardiovascular drift*), necessary to maintain the cardiac output [29–32]. The observed increase in temperature during swimming can affect plasma volume and, consequently, other cardiovascular parameters (mean arterial pressure, heart rate, and stroke volume) [33]. In addition, the increase in the core temperature can reduce the central motor drive [34], which is considered one of the causes of the central origin of fatigue and a limiting factor for performance [35]. Generally, the body thermoregulatory system elicits a shunting of blood to the cutaneous vessels for the dissipation of heat. This mechanism is followed by a reduction in the mean arterial pressure and cardiac volume (i.e., ventricular filling and stroke), and an increase in heart rate. Therefore, it is recommended to drink frequently throughout a triathlon race to minimize the elevation of temperature [29]. In fact, several physiological impairments in multiple systems, organs, and tissues are associated with dehydration and hyperthermia status [36]. Therefore, the consumption of CHO/electrolyte beverages to restore fluids is a strategy to prevent the cardiovascular drift and counteract internal temperature increases, to avoid the worsening performance observed in hypo-hydrated athletes [33, 36].

Hemodynamic parameters also varied during an Olympic distance simulation (1-km swim, 30-km bike, 10-km run) in novice athletes, with an increase in white blood cells (39%), red blood cells (3.8%), and mean cell volume (1%), and a decrease in plasma volume (9.57%), from pre-race to T1 (Table 2), without any change in hemoglobin [27, 28]. The reported elevation in white blood cells after the swim segment could be explained as a *pseudo-leukocytosis* [37–39], which occurs with elevated levels of physical stress. The increase in circulating adrenaline could be explained by the high physical stress condition, which induces the mobilization of white blood cells, with an inverse relationship compared to training level [37–39], and, therefore, with novice athletes having a greater variation in the white blood cells count [26]. In addition, the modifications in red blood cells, as hematocrit and hemoglobin levels, can lead to *sports anemia*, which is known to be caused by the dilution effect of increased plasma volume exceeding the growth of the red cell mass [27].

**Table 2:** Physiological and biomechanical changes during the swim-to-cycle transition

Type	Physiological changes					Biomechanical changes				
	VO_2_/VE/RF during cycling	La/HR after swim/during cycling	Hct/Hb/WBC after swim	RPE / T° after swim	PV after swim	GE during cycling	PW during cycling	SSF/RPM	SSL	PT
FD [Laursen et al.] [18]	+ 5% VO_2_ + 6.7% VE	+ 2% HR					− 4.7%			
HD [Rothschild et al.] [19]	+ 4% VO_2_	+ 4% HR					− 3.8% PW mean			
HD [Rothschild et al.] [41]		HR No Change								
OD [Long et al.] [27]		+ 21.2%LDH + 25% Cre	+ 39% WBC + 3.8% RBC		− 9.6%		− 6%			
OD [Delextrat et al.] [43]	+ 5% VO_2_ + 15.7% VE + 19.9% RF	+ 9.3% HR + 32.2% La				− 13%				
OD [González-Haro et al.] [44]	No change	High to low HR no change					No change			
SD [Kreider et al., 1988] [33]	+ 5.6% VO_2_ + 5.3% VE			+ 0.8% T°		− 17%				
SD [Mc Naughton et al.] [28]					− 3.8% to 4.3%		No change			
SD [Delextrat et al.] [45]	+ 4.5% VO_2_ + 14.4% VE + 15.6% RF	+ 11% HR + 47% La				− 12.1%		+ 14% SSR		
SD [Delextrat et al., 2003] [46]	+ 4.4% VO_2_ + 6.6% VE + 9.4% RF	+ 7% HR + 29.3% La		High RPE		− 4.8%		+ 5.6% RPM		
SD [Delextrat et al.] [55]	+ 5% VO_2_ + 19% VE + 24.8% RF	+ 7% HR + 42.9% La				− 15.5%				
SD [Delextrat et al.] [47]	+ 5% VO_2_	+ 6.4% HR + 16.7% La		High RPE		− 5.4%		+ 5.8% RPM		− 2.9% Pk − 3.9% Mn
SD [Peeling et al.] [48]	No change	+ 75% La		High RPE		− 4.2%	− 9.6%	+ 20.5% SSF		
SD [Peeling et al.] [30]		+ 1.8% HR + 1.2% La		+ 2.4%T°			No change			
SD [Wu et al.] [54]				High RPE			− 6.5%			
SD [Barragán et al.] [49]		+ 59% La					No change	No change RPM		
MTR [Bentley et al.] [50]	No change	+ 3.5% HR ~ + 87% La					− 11% PW mean	+ 18.4% SSF		

*C°* Celsius; Cr: Creatinine; *FD* Full distance; *GE* Gross efficiency; *Hb* Hemoglobin, *Hct* Hematocrit; *HD* Half distance; *HR* Heart rate; *La* Lactate; *LDH* Lactate dehydrogenase; *MTR* Mixed-team relay distance; *OD* Olympic distance; *PT* Pedal torque; *PV* Plasma volume; *PW* Cycling power; *RBC* Red blood cell; *RF* Respiratory frequency; *RPE* Rate of perceived exertion; *RPM* Revolution per minute; *SD* Sprint distance; *SSF* Swim stroke frequency; *SSL* Swim stroke length; *VE* Ventilatory equivalent; *VO*_*2*_ Oxygen uptake kinetics; *WBC* White blood cell

For plasma modifications, an increase in biochemical parameters was found for sodium (1.3% post-swim and 2.6% post-cycle vs. post-swim), lactate dehydrogenase (21.2% post-swim and 10.2% post-cycle vs. post-swim) and creatinine (25% post-swim and 2.9% post-cycle vs. post-swim) (Table 2) [27]. In particular, the increase in creatinine, due to renal blood flow reduction and lactate dehydrogenase, might be related to exercise intensity and duration and athletes’ levels [27, 40]. Furthermore, calcium and aspartate aminotransferase levels after swim significantly increased, as compared to the pre-race values [27].

### Body Temperature

The increase in the rectal temperature (0.8 °C) during the swim segment led to a reduction in mean arterial pressure (9.4%) and in plasma volume [33]. Thermoregulatory mechanisms occur when core temperature increases, with peripheral vasodilation and shunting of blood to the cutaneous vessels for the dissipation of heat. Hence, mean arterial pressure and stroke volume decrease, eliciting an increase in the heart rate during the subsequent cycle segment, required to maintain cardiac output, and an increase in oxygen consumption (VO_2_ = 5.6%) and ventilatory equivalent (5.3%) (Table 2) [33].

The temperature can also increase by wearing a speedsuit, which consists of a one-piece competition suit with channel design and silicon water repellent finish, suitable for use during all three triathlon segments. Indeed, swimming for 750 m at a maximal intensity with a speedsuit tends to increase the core temperature and elevate blood lactate levels (1.2%) and heart rate (1.8%) [30]. Similarly, swimming with a wetsuit during an Olympic distance triathlon test resulted in an increase in the skin (4 °C) and body (1.5 °C) temperature. However, performing the subsequent cycling phase under controlled laboratory conditions (use of a fan) could help to re-establish the normal temperature ranges. Conversely, during regular training and competitions, a hot and humid environment can have a negative impact on performance [40].

### Cardiorespiratory and Metabolic Parameters

During Half and Full distance, the swim segment (longer than 1500 m) has a lower impact on the subsequent cycle segment, and the rate of energy cost is also reduced as compared to the shorter distances events [18, 41]. However, significant differences did not emerge in the cardiorespiratory parameters at T1, even if the heart rate (from 2 to 4%) and VO_2_max (from 4 to 5%) tended to increase, and only the ventilatory equivalent significantly increased (6.7%) during the cycle segment, maybe due to the duration of the session (Table 2) [18, 19, 41, 42]

During an Olympic distance simulation, a previous swim segment (1500 m) induced elevations in blood lactate (32.2%), VO_2_ (5%), heart rate (9.3%), ventilatory equivalent (15.7%), and respiratory frequency (19.9%) at the start of the subsequent cycling exercise compared to the control group, which performed an equivalent cycling session prior to the subsequent cycling exercise [43]. González-Haro et al*.* [44] also found an increase in the blood lactate levels after an Olympic distance swim, but with a tendency to decrease within the 45 min of the cycling session (that lasted 1 h), rising again at the end of the session. However, heart rate at swim-to-cycle transition was not significantly different, while during the cycle segment it tended to significantly increase together with the VO_2_ and ventilatory equivalent [44], differently from Delextrat et al*.* findings [43].

Delextrat and colleagues investigated the Sprint distance performance (750 m swim) and the swim-to-cycle transition, highlighting modifications in the physiological parameters wearing a wetsuit or swimming in drat position. The wetsuit increases buoyancy and reduces hydrodynamic drag, but it can be worn only during the swim segment as its thickness usually limits the execution of running or cycling [45–47]. The authors reported a reduction in heart rate (11%) and blood lactate (47%) after swimming with a wetsuit and, consequently, a decrease in VO_2_ (4.5%), heart rate (3.3%), ventilatory equivalent (14.4%), respiratory frequency (15.6%), and blood lactate (25%) during the cycle segment [45]. Similarly, swimming in the draft position led to a reduction in heart rate and blood lactate after swim and at the start of cycle segment [46, 47]. Moreover, during the cycle segment, VO_2_ (5%), ventilatory equivalent (6.6%), respiratory frequency (9.4%), and RPE (rate of perceived exertion) were lower in the drafting group, showing as swimming in the draft position can lead to lower energy expenditure (Table 2) [46, 47].

Regarding the intensity, during a short event as Sprint distance, swimming with a lower velocity (“S80” from 80 to 85% of the previous swim trial test “STT”) was linked to a better swim-to-cycle performance as compared to swimming at the middle (“S90” from 90 to 95% of the STT) or higher (“S100” from 98 to 102% of the STT) velocity. The highest blood lactate concentrations were found in the S100 group, while the perception of effort was lower in the S80 and S90 groups [48]. Conversely, using a similar methodology of Peeling and colleagues [46], Barragán et al*.* compared intensities of 70%, 80%, and 90% of the maximal swimming speed, obtaining the best performance with the highest intensity combined with an increase in blood lactate concentrations (59%) (Table 2) [49]. Peeling et al*.* [48] also reported an improvement in the cycling gross efficiency (4.2%), that was significantly higher in the S80 group compared to S100 one. During a Mixed-team relay swimming simulation (400 m) before the cycle segment, three different swimming intensities (S90 = 90% of a maximal swimming test; S100 = 100% of max; swim in a drafting position, SDr = 100%), induced variations of the physiological parameters [50]; heart rate (3.5%) and blood lactate (~ 87%) were higher in the S100 group than in S90 one, inducing a great fatigue as also confirmed by the higher perception of effort compared to SDr condition (Table 2) [50]. Notably, it has been also demonstrated that the speed of the first 222 m is a determinant of the overall results, and it is necessary to stay or to catch up with the first cycling group, even though this interpretation of swim is more demanding and causes a higher increase in energy expenditure [7, 23]. Swimming at maximal intensities induces greater fatigue during the cycle segment, probably due to the decrease in muscle blood flow and glycogen storage. The depletion of total or inter-/intra-myofibrillar glycogen volume can impair muscle performance. Indeed, a close relationship exists between high relative workloads and the glycogen storage in the exercising muscles that could limit the capacity during prolonged strenuous work [51]. The working muscle contraction limits the regular blood perfusion in the muscle compartment and slows down the buffering mechanisms of acids [52, 53]. Therefore, the intensity of the effort during the swim segment has a critical impact on the triathlon performance and has to be carefully considered by coaches and athletes during training and competition.

In addition, different swimming pacing strategies can variably affect the Sprint distance performance. A positive pacing—from higher (92% of a 750-m swim time trial “STT”) to lower (73% STT) speed—reduced RPE in athletes [54]. Conversely, the heart rate values measured at the end of the swim performance did not significantly differ in positive pacing as compared to negative one—from lower (73% STT) to higher (92% STT) speed—or even (constant pace at 82.5% STT) [54].

Finally, swimming characteristics can influence physiological parameters. It has been found that swimming with arms only or with arms and legs could induce higher values for cycling blood lactate (42.9%), VO_2_ (5%), heart rate (7%), ventilatory equivalent (19%), and respiratory frequency (24.8%) than those obtained by an isolated cycling performance (Table 2) [55]. Moreover, it has been demonstrated that VO_2_ increased during cycling preceded by high-intensity arm cranking, but not after leg exercise [52].

**Fig. 2:**
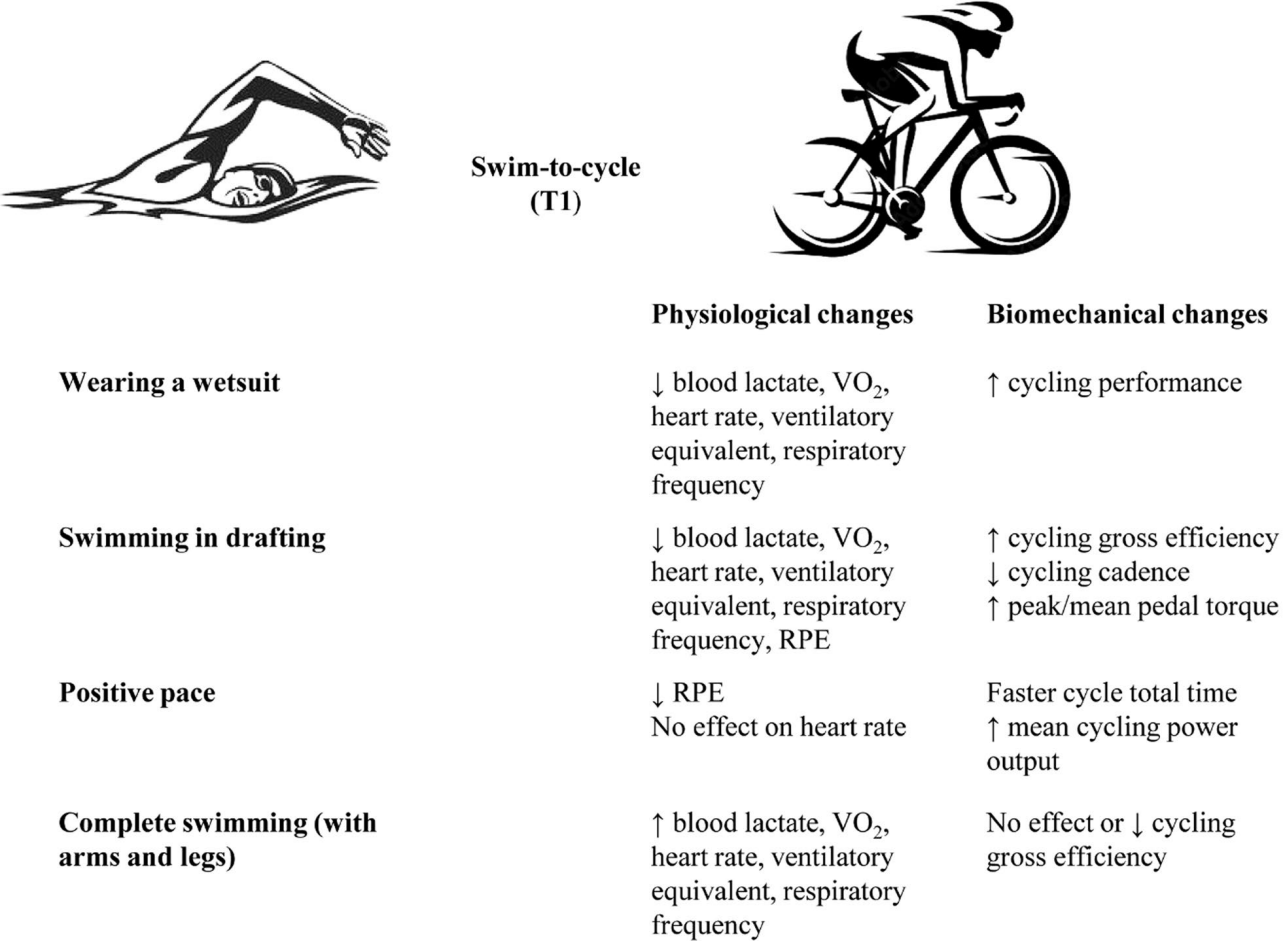
Influence of swim segment on cycle one concerning physiological and biomechanical changes. *RPE* rate of perceived exertion; *T1* swim-to-cycle transition; *VO*_*2*_ oxygen uptake kinetics; ↓: reduction; ↑: increase

Following this evidence on physiological changes, coaches and athletes should consider the importance to maintain the hydration status, by supplementing the CHO and electrolyte and avoiding an excessive increase in core temperature when wearing wetsuit or warming clothes during extreme environmental conditions. The swim pacing strategy is a determinant for the swimming performance, but it can have an impact also on the subsequent cycling performance (Fig. 2).

## Biomechanical Changes

During the T1 transition, several biomechanical parameters might be relevant for the overall performance, such as swim stroke frequency and swim stroke length for the swim segment, cadence (revolution per minute—RPM), gross efficiency, pedal torque, pedal peak, and power output for cycle segment. Similar to physiological parameters, also biomechanical responses can be influenced by race distance, intensity, pacing strategy, and especially wearing the wetsuit.

Generally, the swim stroke frequency increased proportionally (from 14 to 20.5% of the mean value) (Table 2) to the maximal swimming intensity, while a lower swim stroke frequency has been reported at lower intensities [45, 48, 50]. Indeed, swimming for 750 m at 80% of maximal intensity (or wearing a wetsuit) led to a lower stroke frequency compared to swimming at 100% (from 25 to 32 strokes per minute) [45, 48]. Similarly, swimming for 400 m at 90% of maximal intensity compared to 100% highlighted a difference in the stroke frequency (31 vs 37 strokes per minute) [50].

As already stated, the swim segment can influence the subsequent cycle one [2, 4–6]. In fact, during the last meters (200 m) of the swim segment, the athletes tend to increase their speed, activating their legs to express more power in the subsequent cycling, and trying to exit in the first pack [56]. Ortega et al. [57] showed that in the swim-to-cycle transition of short-distance competitions (Sprint and Olympic distance), athletes increased their kick frequency during the last 150–200 m of swim segment, increasing the ratio to 6 beats each stroke and trying to increase the leg blood flow volume. Indeed, during swimming the highest blood flow volume is directed to the upper body. Conversely, the long-distance swim races (Half and Full distance) cause few modifications in the power output during the subsequent cycling performance [18]. Moderate modifications were reported by Rothschild et al*.*, with a decrement in cycling power at 4 mM lactate and in peak power (3.8% and 4.8%, respectively) after a long swim segment [19]. Similarly, total power output decreased by 6% after 2-km swim, without affecting cycling critical power or work done above critical power (Table 2) [41].

During the swim segment of Sprint distance, Peeling et al. [48] demonstrated a variation in swim stroke frequency, according to swim intensity. A decrease in swim stroke frequency (20.5%) emerged for S80 group (80% of max) compared to the S100 group (100% of max), while keeping constant the swim stroke length. Consequently, the gross economy during the cycle segment was higher for the S80 group (4.2%) than S100 one [48]. In accordance with the studies on Sprint distance performance [48, 54], the investigation of Mixed-team relay swim distance (400 m) demonstrated a lower swim stroke frequency (18.4%) in S80 compared to S100, without any significant difference in the swim stroke length. Therefore, during the next cycle segment, the S90 cycling mean power output was higher (11%) than the S100, as confirmed by the constant cycling cadence in the S90 group (Table 2) [50].

As for the physiological parameters, the biomechanical profile of swimming and cycling in Sprint distance performance can be affected by a wetsuit. Athletes can wear the wetsuit in certain situations, with the advantage to save energy, because they reduce drag force [58]. The hydrodynamic drag can be affected by swimming velocity, distance, and kick frequency from the lead swimmer [46]. The speedsuit allowed athletes to swim faster and to reduce fatigue and drag [30]. Furthermore, drafting with wetsuit while swimming leads to a reduction in the passive drag from 16 to 23% during 400 m and was associated with an improvement in swimming time from 3.2 to 5%, compared to swimming without this condition [23]. It has been also demonstrated a lower swim stroke frequency (14%) for the wetsuit group compared to the triathletes without this type of equipment [45]. The reason could be attributed to the increase in the buoyancy with a better water force application and lower drag force, due to a higher development of force in the water for the group with the wetsuit than those without the wetsuit. Moreover, the group without the wetsuit increased the VO_2_ during the cycle segment leading to a lower gross economy (12.1%), compared to the wetsuit group [45]. Finally, the improvement in swimming time (3.2%) observed with a speedsuit was not associated with a concomitant increase in swim stroke frequency and swim stroke length and, subsequently, the cycling performance (Table 2) [30].

The biomechanical parameters also change by swimming in the drafting position with the wetsuit. Indeed, swimming in the drafting position induced an increase in swim stroke length, while swim stroke frequency remained unchanged, demonstrating higher swimming efficiency [23, 59, 60]. Moreover, the cycling gross efficiency in the draft and wetsuit group was higher and the pedal rate was lower compared to the non-draft group [46, 47]. Consistent with the lower cycling cadence, there was also a higher peak pedal torque (2.9%) and mean pedal torque in the draft group (3.9%) (Table 2) [47]. The draft group often adopted 2 beats kicks every arm stroke (therefore, one left kick and one right kick each arm stroke), showing lower cycling cadence with higher peak pedal torque, whereas the non-draft group, adopting 6 beats each stroke, showed higher cadence [47]. It could be hypothesized an involvement of the post-activation potentiation phenomenon, which refers to the increase in muscle contractile responses due to a prior muscle activity, causing a subsequent increase in the muscular contraction, with the swim segment acting as conditioning for the subsequent cycle segment [61, 62]. Therefore, it could be hypothesized that a high kick rate might generate the post-activation potentiation and lead to similar effects on cycling performance in terms of high pedal rate [61]. However, the understanding of the effect of the kick rate on the physiological parameters during the cycle segment and the activation of the post-activation potentiation is not fully explained. Therefore, further studies are encouraged to explore an optimal swim kick rate during the swim segment that could positively influence the subsequent cycle segment, and the effect of the training program on swim kick rate.

Furthermore, with the manipulation of the swimming pacing strategy, the final race result can be augmented by a positive pace [54]. During a Sprint distance performance of 750 m, the positive swim pace led to a faster cycling total time (4%), a higher average cycling power output (6.5%), and a lower RPE (Table 2) [54].

Swimming characteristics (i.e., swimming with arms only, legs only, or complete swimming) did not seem to affect the pedal rate, even though authors reported a reduction in the cycling gross efficacy (15.5%) in swimming with arms only or complete swimming [55]. The gross efficiency decrement during the cycling performance after the swim segment can be linked to the muscle mass involved during swimming with arms only or complete swimming, and consequently to the higher energy expenditure required. Indeed, higher values for heart rate, VO_2_, ventilatory equivalent, and respiratory frequency were found after swim, especially after complete swim segment [55].

As highlighted above, coaches and athletes should consider the importance of swim stroke frequency and length during the swim-to-cycle training. Indeed, swimming in the drafting position and wearing a speedsuit or wetsuit lead to a better swimming time with a higher swim stroke length and a reduced drag force, preserving the energy for the subsequent cycle segment. Similarly, the management of swim kicks frequency might be essential for the subsequent cycle segment (Fig. 2).

## The Interlink Among Physiological and Biomechanical Responses

Limited evidence is available for the possible interlink among physiological and biomechanical responses during the swim-to-cycle transition. Relationships between VO_2_ (i.e., energy cost) and cycling cadence have been evaluated to determine the lower energy cost at a specific pedal rate (i.e., energetically optimal cadence), demonstrating a range value from 73 to 86 RPM [26, 63]. However, the optimal pedaling cadence depends on the level and type of athlete (cyclists have a more efficient pedaling cadence with higher RPM compared to triathletes) [64]. Although the second transition (cycle-to-run) is not part of the current review, and has been debated elsewhere [20], it is worth mentioning that a low cadence during the cycle segment could lead to a lower stride frequency during the subsequent run segment [63]. Moreover, the energy cost of Olympic distance swimming (1500 m) caused a decrease in the cycling gross efficiency (13%) [44]. Similarly, an increase in rectal temperature during the prior swim segment induced a decrement in gross efficiency (17%) during the cycle segment [33].

Focusing on the biomechanical parameters, the swim stroke frequency and the higher swimming intensity induced higher blood lactate concentrations, also reported in the cycle segment, due to a higher pedaling cadence [45–48, 50]. In both swim and cycle segments, the elevation in blood lactate concentration could be attributed to the recruitment of different and additional fibers types [48]. To satisfy the high ATP demand in the fast twitch muscle fibers, the glycolytic metabolism increases [48] because of a greater recruitment of type II muscle fibers [31], together with a greater blood lactate production [43].

It is worth mentioning the importance of swimming pool length (25 vs. 50 m) when physiological and biomechanical parameters are investigated to avoid misinterpretation among studies. In fact, swimming in a 25 m pool resulted in extra turns and it induced a higher swim stroke length and a lower blood lactate concentration and heart rate compared to swimming in a 50-m pool, generating a more efficient overall performance [44].

The limited research available with the combined evaluation of physiological and biomechanical parameters during the swim-to-cycle transition does not allow to make definite conclusions on the possible associations among physiological and biomechanical parameters. Therefore, it is recommended to design an intervention program aimed at exploring the impact of different strategies on both physiological and biomechanical parameters during both swim and cycle segments.

## Practical Implications for Triathletes

The available evidence on the physiological and biomechanical parameters could be translated into some practical implications for coaches and athletes and applied during training and competition, even though the triathlon performance still deserves further investigations, particularly for the swim-to-cycle transition. Firstly, it is important to emphasize the need to propose periodic swim-to-cycle tests for the evaluation of physiological (i.e., heart rate, blood lactate, VO_2,_ ventilatory equivalent, respiratory frequency) and biomechanical parameters (i.e., gross efficacy, swim stroke frequency and length, power output, cadence) and for the determination of an optimal interlink among them. As a consequence, intensity and strategy during training and competition could be determined. In light of the current knowledge, more implications could be derived for the short-distance triathlon (i.e., Sprint distance and Mixed-team relay). Considering the swimming segment, training intensity should be maintained around 80–90% of a previous maximal swim test, while a race swimming simulation should consider the selection of a positive pacing strategy from 92 to 73% of a swim time trial. Moreover, for the Mixed-team relay, the swim segment should be trained with the maximal intensity during the first 200 m, and the use of drafting position and wetsuit is recommended because of the lower energy requirement. A practical approach for the monitoring of the internal training load and the evaluation of training intensity could be the use of the perception of effort (RPE scale), possibly associated with blood lactate measurements. Regarding the cycle segment, training could consider the adoption of a different cycling cadence based on the training planning and athlete level. If only swim and cycle segments are part of the training program, athletes could adopt a lower cadence compared to that used in the cycle-to-run training. Moreover, it is recommended to empower the strategy to express higher power in order to stay in the first pack. In the case of combination of the three segments, a constant and high cycling cadence would be beneficial for the subsequent run segment.

Moreover, the importance of strength training for endurance sports such as triathlon is well documented. In this regard, a recent meta-analysis demonstrated the greater beneficial effect of non-sport-specific training aimed at improving maximal strength in triathletes compared to other athletes, with an improvement in endurance performance [65, 66].

## Future Directions

The swim-to-cycle transition is not fully explained, particularly for the potential impact of the swim segment on cycle one. Therefore, future studies could better elucidate whether different swimming modalities, having an active legs participation, might positively influence the following cycling performance. Considering the post-activation potentiation phenomenon, it could be hypothesized that swimming with high kick rate could activate the legs in the subsequent cycle segment with a more efficient expression of power. In particular, training intervention could be proposed to test the hypothesis of training adaptations on swim kick rate and their effects on cycling performance. However, for a better understanding of the factors associated with triathlon performance it is recommended to adopt an experimental approach consisting of a combined measurement of physiological and biomechanical parameters.

## Conclusions

The complexity of triathlon performance, consisting of three different disciplines and different race distances, does not allow to generalize findings from every study regarding the swim-to-cycle transition, but every evidence is limited to the specific race distance. The influence of the swimming interpretation on the subsequent cycle segment is more evident for the short-distance competitions (Olympic and Sprint distance, and Mixed-team relay) compared to the long events (Full distance). Several changes in physiological and biomechanical variables emerged during the swim-to-cycle transition, considering also the level of athletes, with higher modifications for those at a lower level. The higher increase in blood lactate when the intensity of swimming is maintained high is related to a lower cycling power output and a greater oxygen consumption. The increase in the swim leg frequency during the last meters of the segment seems to be necessary to stay within the first cycling group, leading to a similar effect on cycling performance in terms of high cycling cadence, which, in turn, can be beneficial for the next run segment. Moreover, the increase in swim leg frequency in the last part of swim segment is aimed to increase leg blood flow volume. Wearing a wetsuit or swimming in the drafting position may preserve energy for the following cycle segment. The increase in swim efficiency (hydrodynamic) is achieved with a lower swim stroke frequency, while an improvement in cycling economy is obtained with a higher pedal torque. Moreover, keeping a swimming intensity around 80–90% and the use of a positive pacing strategy are conducive to a better performance.

## Data Availability

Not applicable.

## Abbreviations

ATP: Adenosine triphosphate
Cre: Creatinine
C°: Celsius
FD: Full distance
GE: Gross efficiency
Hb: Hemoglobin
Hct: Hematocrit
HD: Half distance
HR: Heart rate
La: Lactate
LDH: Lactate dehydrogenase
Mn: Mean
MTR: Mixed-team relay distance
OD: Olympic distance
Pk: Peak
PT: Pedal torque
PV: Plasma volume
PW: Cycling power
RBC: Red blood cell
RF: Respiratory frequency
RPE: Rate of perceived exertion
RPM: Revolution per minute
SD: Sprint distance
SSF: Swim stroke frequency
SSL: Swim stroke length
STT: Swim trial test
T°: Core temperature
VE: Ventilatory equivalent
VO_2_: Oxygen uptake kinetics
WBC: White blood cell
